# Automated Quantum Dots Purification via Solid Phase Extraction

**DOI:** 10.3390/nano12121983

**Published:** 2022-06-09

**Authors:** Malín G. Lüdicke, Jana Hildebrandt, Christoph Schindler, Ralph A. Sperling, Michael Maskos

**Affiliations:** 1 Fraunhofer Institute for Microengineering and Microsystems IMM, 55129 Mainz, Germany; jana.hildebrandt@bam.de (J.H.); c.schindler@interbran.de (C.S.); michael.maskos@imm.fraunhofer.de (M.M.); 2 Federal Institute for Materials Research and Testing, 12205 Berlin, Germany; 3 Interbran Advanced Materials GmbH, 76684 Oestringen, Germany

**Keywords:** quantum dots, purification, solid phase extraction, flow chemistry

## Abstract

The separation of colloidal nanocrystals from their original synthesis medium is an essential process step towards their application, however, the costs on a preparative scale are still a constraint. A new combination of approaches for the purification of hydrophobic Quantum Dots is presented, resulting in an efficient scalable process in regard to time and solvent consumption, using common laboratory equipment and low-cost materials. The procedure is based on a combination of solvent-induced adhesion and solid phase extraction. The platform allows the transition from manual handling towards automation, yielding an overall purification performance similar to one conventional batch precipitation/centrifugation step, which was investigated by thermogravimetry and gas chromatography. The distinct miscibility gaps between surfactants used as nanoparticle capping agents, original and extraction medium are clarified by their phase diagrams, which confirmed the outcome of the flow chemistry process. Furthermore, the solubility behavior of the Quantum Dots is put into context with the Hansen solubility parameters framework to reasonably decide upon appropriate solvent types.

## 1. Introduction

The appeal of luminescent nanoparticles, Quantum Dots (*QDs*) in particular, is due to the high-value technical applications. Besides fundamental research, they have been successfully integrated in display production at a commercial level [1] and as a source candidate for quantum communication technology on a research level [2]. In contrast to organic chromophores, they show higher photochemical stability, narrower optical emission spectra, larger molar absorption coefficients, and the ability to tune the emission wavelength by the size of the nanoparticle, which are the decisive reasons the synthesis of inorganic *QDs* advanced from lab scale batch synthesis towards process engineering practice [3]. In this context, the usage of continuous micro-flow reactors has proven to be a method for reproducible production of many semiconductor nanoparticles systems with high quality [4,5,6]. The next stage towards integration of this material into a new matrix or subsequent synthesis protocols is its purification. The down-streaming process for composite materials on a preparative scale are still cost-intensive because often technical approaches like liquid chromatography for molecular species cannot be transferred with the same efficiency to colloidal particles [7,8]. Three principles for the separation of the desired colloids and the excess synthesis components are recognized: distribution in size [9], polarity [10], and electrophoretic mobility [11]. Perturbating the dispersion of colloids by adding a weak solvent leads to agglomeration and precipitation of the nanoparticles. The solid cake can be redispersed in suitable solvents afterwards, after taking off the supernatant containing the molecular species present in the original medium, resulting in a purified product. Centrifugation accelerates the sedimentation of these agglomerates, thus precipitation and redispersion (P/R) is conventionally used for lab scale purification of samples. Although centrifuges are available in most laboratories, for upscaling more manual handling is required, while semi-continuous centrifuges are more cost-intensive. In addition, this method can negatively affect the dispersion and fluorescent properties of *QDs*, due to the mutual interaction at the nanoparticle surface [12]. Capping agents at the surface are compulsory for providing colloidal stability but also essential to diminish non-radiative decay after optical excitation. Bonding simulation studies conclude that binding states of under-coordinated surface atoms intrude the band gap, deteriorating the emission properties. In this context, the redox potential of the surface atoms determines the course for re-establishing charge neutrality after the displacement of former bonding partners [13].

On a phenomenological level, the fluorescent intensity depends on the total ligand molecule concentration, as shown for Lewis acids as coordinating bonding partners of *QDs* with zinc blende crystal structures [12,14]. Furthermore, NMR studies revealed the solubility dynamics for one common ligand group of CdSe [15,16]. Carboxylates bind to the Cd-site at the nanoparticle surface [17]. However, exchange reactions occur both with aprotic as well as protic organic solvents as a function of permittivity or acidity, respectively. These experimental and theoretical findings can be summarized as a trade-off for the down-streaming process between sufficiently removing components from the original medium, as needed for the subsequent process steps. At the same time, enough surface ligand molecules should be maintained in solution, considering their solubility equilibrium to densely cover the particle surface, to avoid trapping states.

Some recent studies demonstrated technical approaches in *QDs* purification with organic solvents included in order to meet the goals at a semi-preparative scale. Using gel permeation chromatography, the excess ligand concentration serving as marker was decreased below the detection limit of its ^31^P-NMR signal [18]. A P/R-step is compulsory beforehand due to irreversible fouling of the column material. The same researchers developed a continuous extraction setup including multiple phase separation membranes [19]. In order to reach the operational window, a dilution of the octane-based synthesis solution prior to purification is necessary for this method. In contrast to the previous separation principles, Woo et al. acquired parameter settings for free-flow electrophoresis in micro-channels [11], trapping up to 87% of the original *QDs* at porous electrodes at high voltage up to 500 V. This approach could reduce the excess of trioctylphosphine below the detection limit via a subsequent washing step.

In the current article, a solid phase extraction (SPE) procedure is proposed as a facile alternative with lower material costs that is able to directly process the crude reaction mixture. For this purpose, common components for the synthesis of CdSe/ZnS system are used: the solvent 1-octadecene (ODE), excess of ligand molecules, trioctylphosphine (TOP), oleylamine (OLA), oleic acid (OA), and excess of the shell precursor zinc diethyldithiocarbamate (ZDEC). This combination serves as realistic model recipe for *QDs* synthesis and takes into account the complexity of mutual miscibility. Solid phase extraction exploits the variable affinity of synthesis components towards a stationary phase and the difference of distribution coefficients between original and extraction medium, while providing a high surface to volume ratio. It is predestined to be transferred towards a continuous process, which accelerates and automates manual handling. In order to gain a broader scope of the mutual physicochemical interactions, Hansen solubility parameters (HSP) of CdSe/ZnS *QDs* were investigated in order to classify solvents for the purification scheme. These physicochemical parameters allow a characterization of compatibilities between substances based on the thermodynamics of three molecular interaction energies (intermolecular polar forces, hydrogen bonding, and dispersion forces). Together they span a quantitative three-dimensional parameter space. A small vector distance between two chemicals indicates a high compatibility, a large vector distance indicates the opposite. Since the proof of concept in the 1960s, a lot of substances have been added into the database [20]. Originally, Charles Hansen extended an existing concept to dispense polymers in solvent blends by turning two non-solvents into a useful solvent at a calculated volume ratio [21]. In the following, the HSP approach has been transferred to characterize and even to predict solubility between arbitrary solids and solvents [20]. Recently, the HSP concept is evolving with respect to colloids. In this context, Segets et al. proposed the detection of the cloudiness within an analytical centrifugation as a routine to determine the boundary of the compatibility zone [22]. Meanwhile, calling it dispersibility or solubility with respect to nanoparticles is still an open debate within the HSP research community [23,24].

Here, we show that Dynamic Light Scattering (DLS) is also an effective technique to assess the distinct transition point from fully dispersed to agglomerated nanoparticles within the HSP space next to visual inspection of test tubes.

## 2. Materials and Methods

### 2.1. Continuous Synthesis of CdSe/ZnS Core/Shell Nanoparticles

For synthesis of CdSe/ZnS *Quantum Dots* (*QDs*), cadmium oxide powder (99.99%), selenium powder (99.95), 1-octadecene (90%), oleic acid (65–88%), oleylamine (70%), trioctylphosphine (97%), and zinc diethyldithiocarbamate (95%) were purchased from Strem Chemicals, Inc. (Newburyport, MA, USA), Acros Organics (Fisher Scientific International Inc., Pittsburgh, PA, USA), Acros Organics, PanReac AppliChem (Chicago, IL, USA), J&K Scientific (San Jose, CA, USA) and abcr GmbH (Karlsruhe, Germany), respectively. All synthesis educts were used as received and stored under inert gas conditions.

**Figure 1:**
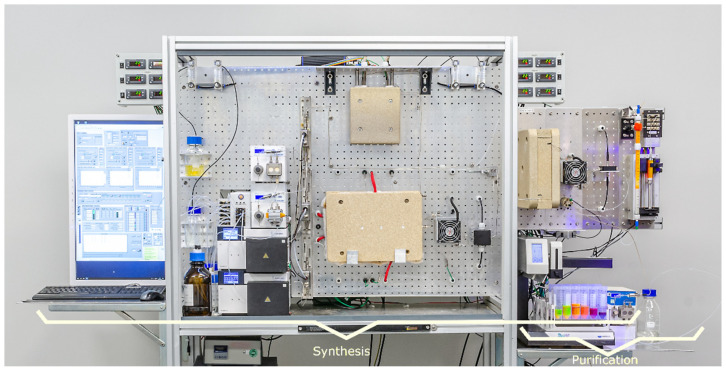
Manufacturing platform for continuous synthesis and purification of CdSe/ZnS Quantum Dots.

CdSe/ZnS core-shell nanoparticles were synthesized continuously in one step within a microfluidic tube reactor, as previously mentioned in [25,26]. The modular, computer-integrated manufacturing setup is shown in Figure 1. An overview is given by a flow chart in Figure S1. Three precursor solutions, stored in inert gas, are injected into the system by 10 mL piston pumps (Knauer 40P, Berlin, Germany). Mass flow of the educt streams is monitored using mass flow controllers (mini Cori-Flow/Bronkhorst, Gelderland, The Netherlands), guaranteeing a constant flow rate ratio between both core and shell-precursor solutions of 1:1 and 1:7, respectively. Within the first heating zone (315X-stainless steel tube, 0.25 m, inner diameter = 0.5 mm embedded between two aluminum heating plates) the metalorganic precursors decompose at 300 °C into their monomers. In the course of the reaction scheme, nucleation and subsequent growth of CdSe core particles occurs according to the residence time, here 2.5 s. Homogenous mixing of the first two precursors is accomplished within an interdigital micro mixer, developed and manufactured at Fraunhofer IMM (Mainz, Germany), as displayed within Figure S2 [27,28,29]. In this context, the multilaminar flow profile is realized by the inlet microstructure and the refocusing of streams by the steel case. A heat exchanger performs quenching of the first reaction solution. The first absorbance maximum of CdSe-core particles is detected in-line within a flow-cell via a CCD spectrometer with a resolution of approx. 0.6 nm (ULS2048L-RS-USB2 from Avantes, Apeldoorn, The Netherlands). A fiber-optic deuterium-halogen lamp (Avantes DHL-Bal) is used as the light source. Overall spectral information between 320 and 650 nm is recorded periodically with 1 Hz acquisition rate. Shell growth is performed by mixing the core solution with a monomolecular precursor at room temperature within a caterpillar mixer developed and manufactured at Fraunhofer IMM (Mainz, Germany) (channels of 300 μm, 6 crossing points), as displayed in Figure S2. The reaction takes place in the second heating zone at 130 °C for 80 s (315X-stainless-steel tube, 10 m, inner diameter = 1 mm embedded between two aluminum heating plates). After quenching, the reaction solution the Stokes shift of the first absorbance maximum is detected as well as the fluorescence emission within two subsequent flow cells. The latter is realized in 90° geometry using a UV-LED emitting at 365 nm. Finally, a fraction collector switches between product and waste production with a delay of two mean residence times by default. The individual components of the synthesis setup are controlled by a LabVIEW program, which also visualizes and records all process data. An absorbance and emission spectrum of the CdSe/ZnS *QDs* used throughout all purification studies is illustrated in Figure S3. The results of a thermogravimetric analysis of the stock solution are provided in Figure S4, showing three significant steps of mass loss starting from 240 °C.

Transferring Se, CdO, and zincdiethyldithiocarbamate into soluble metallorganic precursors took place before the continuous synthesis at inert gas conditions. For this purpose, 44 mmol selenium powder is dissolved with trioctylphoshpine (1:1.7 equivalent) within 1-octadecene (1:12 equivalent) at room temperature (c_0_ = 220 mmol L^−1^). Cd-oleate is realized by complexing 40 mmol CdO with oleic acid (1:4 equivalent) within 1-octadecene (1:12 equivalent) at elevated temperatures (120 °C for 1 h, 210 °C for 3 h), resulting in a honey-colored solution. ZnS shell material can be generated in-situ by decomposing the monomolecular precursor. For this purpose, 280 mmol zincdiethyldithiocarbamate was mixed with oleylamine (1:2 equivalent) and trioctylphosphine (1:1.4 equivalent) within 1-octacedene (1:7 equivalent) at room temperature.

### 2.2. Purification of CdSe/ZnS Core/Shell Nanoparticles

#### 2.2.1. Derivatization of SiO_2_ Beads

As sorbent materials, two different size ranges of polished soda-lime silica beads were used. Their size ranges from 70–100 and 250–500 μm, respectively, according to the supplier Sigmund Lindner (Warmensteinach, Germany). 100 g was cleaned in 0.5 vol% Hellmanex III solution and washed in deionized water. Afterwards, they were covered with 1M HCl solution and agitated overnight in order to prepare the glass surface for the subsequent liquid silanization reaction scheme. They were washed with deionized water until a pH value of 7 was reached, and dried at 120 °C overnight before being re-dispersed in 300 mL n-hexane. For silanization, octadecyltrimethoxysilane (OTMS) or 1H,1H,2H,2H-Perfluoroctyl-trichlorsilane (PFOCTS) was added at room temperature on the basis of previously reported reaction schemes [30]. The glass beads were agitated for 5 days before being washed with excess n-hexane and treated at 80 °C overnight. With respect to volume fractions, the minimum number of reagents was calculated considering a total bead surface area of closely packed equal spheres and a molecular surface coverage of 0.3 nm^2^ for OTMS and PFOCTS, as estimated via XPS analysis for octadecyldimethylmethoxysilane [31]. Additionally, the minimum amount was scaled by a 500-fold. Hence, for 100 g of 70–100 μm beads, 8.83 g OTMS or 7.71 g PFOCTS was provided. The bead morphology before and after silanization is shown in Figure S5.

#### 2.2.2. Purification Platform

For solid phase extraction experiments of synthesized CdSe/ZnS-*QDs* ethanol (99.7%), n-hexane (97%) and methanol (99.9%) were purchased from VWR chemicals (Avantor, Radnor, PA, USA) and Carl Roth GmbH & Co. KG (Karlsruhe, Germany). The automated experimental setup for the down-streaming process of CdSe/ZnS *QDs* is shown in Figure 1. It includes a syringe pump (Nexus N6000/Chemyx, Inc., Stafford, TX, USA) for the *QDs* stock solution and three piston pumps for adhesion, washing, and elution solvent (Knauer 40P, Berlin, Germany), as well as 3-way valves (Bürkert, Ingelfingen, Germany/Type: 6724 T 1,2 FFKM Peek 24V) to load the packed column sequentially from bottom to top. Flow rates are regulated by mass flow controllers (mini Cori-Flow/Bronkhorst, Gelderland, The Netherlands). Fluorinated ethylene-propylene tubes (inner diameter = 0.75 mm) are applied as connecting pieces between pumps, valves, column, and the spectral flow cells for in-line analysis in transmission and emission geometry. By means of a fraction collector, washed and purified *QDs*-solutions can be separated as soon as a critical fluorescence signal is detected on-line. Volatile washing solvents were eliminated via heat treatment at 100 °C for 14 h since TGA analysis of the raw solution does not show a significant mass reduction until 240 °C as illustrated in Figure S4. The residual impurities are shown exemplary in Figure S6. Their mass determines the purification score in relation to the original mass of 2 mL stock volume. As a stationary phase, a borosilicate glass column (006BCC-10-10-FF) from Diba Omnifit Labware (BGB Analytik, Böckten, Switzerland) was integrated, which is demonstrated exemplarily in Figure S7. For standard settings, a 330 mm long column (inner diameter = 7.85 mm) was used. 23.6 mL column volume was created after capping both ends with PEEK thread nuts and PTFE-frits with nominal pore sizes of 10 μm according to the manufacturer. Surface-modified SiO_2_ beads were transferred as a slurry into the column. In order to compress and pack the stationary phase tightly, maximal flow rates of 10 mL min^−1^ were applied for 5 min.

#### 2.2.3. Residence Time Distribution (V_E_)

The mean residence time distribution (E(t)) for the entire purification set up, including the 330 mm long column filled with 70–110 μm SiO_2_ beads, was estimated via pulse experiments from the fluorescence signal intensity of 21 mM Rhodamine 6G in methanol as tracer material, as shown in Figure S8. Mean residence time (t_m_) and its variance (σ) was determined according to the Equations (S1)–(S4). In this context, the integral was discretized as a sum integral. The effective elution volume along the flow path was estimated from the mean residence time and extended according to the observed tailing once 99.5% (F(t)) was reached. It is denoted as the elution volume (V_E_) within the main text.

### 2.3. Physicochemical Characterization

#### 2.3.1. Photospectroscopical Determination of *QDs* Concentration and Fluorescence Intensity (QY)

Off-line optical spectroscopy was performed with a Cary 50-Scan for UV/Vis spectroscopy and Cary Eclipse fluorescence spectrometer from Agilent Technologies (Santa Clara, CA, USA).

Photoluminescence quantum yields were determined according to Equation (S4) following a recommended procedure [32]. As an external standard, Rhodamine 6G was used (99.99% from Sigma Aldrich, St. Louis, MI, USA) in isopropanol (99.99% from Sigma Aldrich) with a quantum yield of 95%. As error estimation, confidence intervals of both linear regression slopes shown in Figure S9 were determined as part of the regression analysis according to L. Sachs [33]. In this context, the confidence interval of the slope β = ±4% for the standard and β = ±1 − 15% for the purified *QDs* samples were calculated with α = 0.05 using the Student’s *t*-distribution. Averaging the combination of maximal lower and upper slope errors of both standard and sample fit leads to the quantum yield deviation error.

Particle concentrations were determined using the law of Lambert and Beer. As molar extinction coefficient, the empirical correlation proposed by Mulvaney et al. was applied, which interrelates the first absorption peak wavelength with the molar extinction coefficient and thus the particle concentration [34]. In this context, the absorption shift resulting from the shell material ZnS was equated with core material since the relative concentration before and after the down-streaming process is of prime importance.

#### 2.3.2. Gas Chromatography and Mass Spectroscopy Analysis (GC/MS)

For GC/MS analysis, the system GCMS-QP2010 from Shimadzu (Kyoto, Japan) was used equipped with the AOC-20i/AOC-20s-autosampler, a 30 m × 0.25 mm × 0.25 μm Zebron ZB-5MSi-column, and a single-quadrupole mass spectrometer.

For GC/MS analysis the supernatants of the *QDs* stock and purified solution were examined. For this purpose, stock solution was diluted with n-hexane using four times the amount of the original medium, while the anti-solvent ethanol was added directly in case of the purified samples using twice the resulting volume amount. After sedimentation of the agglomerated nanoparticles within a centrifuge at 4500 rpm for 15 min (rotor Ø 50 cm), the supernatants were analyzed spectroscopically for absence of fluorescent nanoparticles. Otherwise, further anti-solvent was added, as in the case of the stock solution. In order to sharpen elution fronts of oleylamine and oleic acid, the derivatization agents acetone and N-methyl-N-(trimethylsilyl)trifluoroacetamide (MSTSA) were added to generate an imine and oleatesilylether instead. Acetone was introduced as a diluting solvent and MSTSA was injected into the measuring vial one day before analysis at room temperature. The molar excess of the derivatization agents equal 500 and 1000 with respect to the maximal number of substances expected within the stock solution for oleylamine and oleic acid in case of incomplete yield during the synthesis. Calibration curves and the location of sample concentrations within the calibration region are displayed in Figure S10, including decomposition products like trioctylphosphine oxide, since samples were stored at ambient conditions.

The standard GC/MS method includes a split injection temperature at 250 °C and a temperature profile within the column from 180 to 330 °C ramping in 10 K min^−1^, while the ion source temperature is set to 230 °C. Holding the column at 330 °C for 15 min at the end results in a total program time of 31 min.

#### 2.3.3. Thermogravimetric Measurements (TGA)

TGA/DSC were conducted within 1 LF/1536 Pen from Mettler Toledo within an alumina crucible in a temperature range of from 30 to 1000 °C (10 K min^−1^) with a dry air flow of 40 mL min^−1^.

#### 2.3.4. Scanning Electron Microscope (SEM)

For visualization of the sorbent material, the scanning electron microscope Zeiss LEO 1550 VP (Zeiss Group, Oberkochen, Germany) was used with an anode voltage of 5 kV and working distances of 3.8 or 5 mm, respectively, as indicated. Samples were sputtered with an Au layer for 30 s with an approximate rate of 0.5 nm/s via a Balzers MED 010 sputter coater beforehand.

#### 2.3.5. Transmission Electron Microscopy (TEM)

For visualization of the *QDs* after the purification process, the obtained solution was diluted in Toluol with a dilution factor of 100 in total and dried on carbon-coated copper grids. TEM measurements were performed with a Zeiss Libra 120 electron microscope (Zeiss, Oberkochen, Germany) at 120 kV acceleration voltage. The images were taken by a CCD camera.

### 2.4. Contact Angle

For visualization of contact angles within the three-phase system, specimen slides were treated in the same manner as the beads. Within a glass container containing ODE-saturated solutions of ACN, MeOH, or EtOH, droplets of ODE with and without 3 vol% OLA were placed on the specimen slides using a needle with an outer diameter of 1.27 mm. The latter was appropriate to obtain surface tensions expected from water and ODE within the 2-phase system against atmospheric conditions at 22 °C [35]. Equilibrium state was expected after waiting 5 min.

### 2.5. Hansen Solubility Parameter Analysis

The list of test solvents used for the determination of the Hansen solubility parameters, their classification and their purities respectively is given in Table S1.

#### Dynamic Light Scattering (DLS)

Dynamic Light Scattering spectra were obtained with the Zetasizer Ultra from Malvern Panalytical (Malvern, UK) at a constant temperature of 25 °C. In this context, solutions of 1 mg purified *QDs* in toluene (99.99%, anhydrous, filtered via 0.02 μm PTFE) and propylene carbonate (99.99%, anhydrous, filtered via 0.02 μm PTFE) were measured in a back-scattering mode. The optimal measurement position was assessed iteratively according to the autocorrelation fit statistics, which did not exceed an error above 1.3%. The known volume contraction of the solvent blend was neglected since the maximal deviation is below 1% [36].

### 2.6. Analysis of Variance (ANOVA)

The design of experiments (fractional factorial 2^4−1^ split design including one repetition of each factor settings) and the ANOVA analysis of the key indicators were conducted using the Design Expert software package (Stat-Ease Incorporation: Minneapolis, MN, USA, version #12) [37]. In this context, Pareto analysis and normal probability plots were consulted preliminarily to decide upon inclusion of the factors and their mutual interactions within the linear regression model.

## 3. Results and Discussion

Since solid phase extraction is based on balancing solubility and affinity along the column bed, it is comparable to affinity chromatography. The adhesion of excess ligand molecules to the sorbent material in combination with the inherently strong interaction of the inorganic particle surface with these ligands results in an immobilization of both species. In contrast, the residual solvent mixture, consisting of the weak solvent and synthesis components, passes the column and becomes eluted. In the following step, when a second volume of washing solvent is applied, excess ligand groups are increasingly removed, while the *QDs* remain trapped. Finally, when switching to a good solvent, the *QDs* with a fraction of remaining ligand molecules can be eluted and recollected. Consequently, the purification scheme involves four consecutive steps:

- Conditioning of the solid phase column with weak solvent
- Injection of crude product, i.e., *QDs* synthesis mixture and additional weak solvent, causing adhesion
- Injection of extraction solvent
- Elution of purified *QDs* via strong solvents.

**Figure 2:**
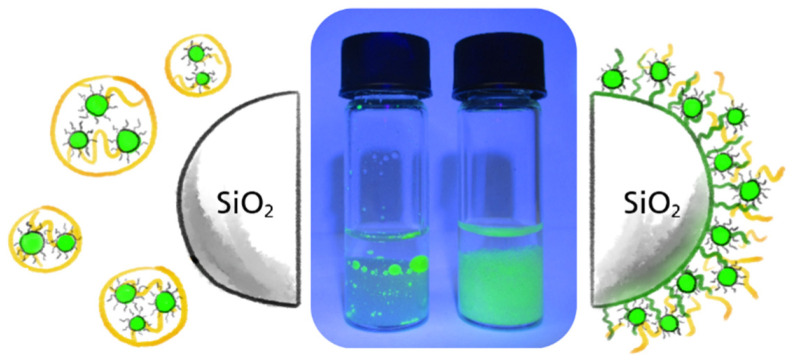
Raw synthesis solution in contact with MeOH and untreated non-porous SiO_2_-beads (**left**) or hydrocarbon surface-modified SiO_2_-beads (**right**).

**Figure 3:**
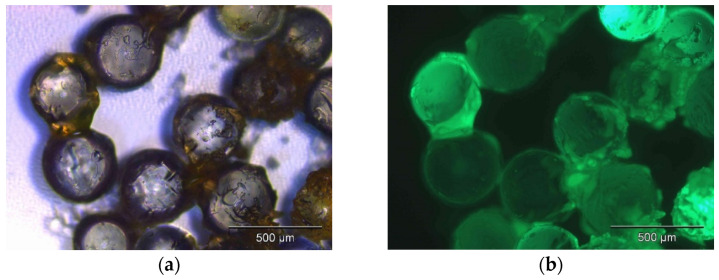
Bright-field (**a**) and fluorescence microscopy (**b**) of *QDs* adsorbed hydrocarbon surface-modified SiO_2_-beads.

First, a weak solvent was used to condition the column material before the crude concentrated synthesis solution was injected in the second step. During this step, mutual interaction can be observed between synthesis components to solvent and synthesis components to sorbent, respectively. Looking more closely, distinct cases exist for the whole system containing oil, surfactants, nanoparticles, and a weak solvent. An immiscible two-phase liquid system was produced in the case of, e.g., methanol or the nanoparticles agglomerate in the homogenous mixed phase in the case of, e.g., ethanol. A classification of further weak solvents leading to the one or the other state is given in the context of the HSP study within Section 3.2.2. Introducing the sorbent material yields to a three-phase system in which the interfaces re-arrange to minimize the total interfacial energy. This leads to wetting and subsequent adhesion of the oil phase, including of the fluorescent particles, to the column bed. A juxtaposition of two different morphologies of the system after the second process step is shown in Figure 2 with untreated sorbent and non-wetting emulsion, in contrast to surface-modified sorbent material. Microscopic images of latter state show fluorescent particles embedded within a gel matrix adhering to the glass beads (See Figure 3). This means that the *QDs* result in being in contact with their native environment on the solid phase substrate, while most of the continuous phase can be separated. The suggested approach is presumably more gentle as compared to nanoparticles with direct contact to the anti-solvent, leading to their agglomeration.

In contrast to common stationary phase materials, non-porous SiO_2_ beads with modified surfaces were generated by using alkyl- and perfluoroalkylsilane, respectively. A comparison between both surface modifications is presented by preliminary batch tests in Section 3.1. and is evaluated in context with the continuous process scheme in Section 3.2.1. The modified beads exhibit the advantages of being inert, compatible with organic solvents, without porosity and hence robust for rapid conditioning of the column in order to realize upscaling of purification. Trapping and spreading the *QDs* on the solid phase allows effective separation of components of the original organic phase, enhanced via a subsequent extraction step. Protic and aprotic candidates are compared in terms of their efficiency in Section 3.2.1. The column capacity for the *QDs* is derived from the height of their retention on the column bed and did not exceed 76%. Finally, the purified *QDs* can be eluted with strong solvent, such as n-hexane. Alternative strong solvents are listed in the context of the HSP study in Section 3.2.2. The elution is monitored by UV-vis spectroscopy. The resulting chromatogram after one purification cycle is demonstrated in Figure S11, showing an increase and decrease of the signal as the original synthesis matrix is separated.

### 3.1. Batch Assessment

Adhesion is a system state stemming from the characteristics of all of its components. Both the surface characteristics of the sorbent material, as well as the intrinsic interfacial tension between the continuous phase and the weak solvent, determine the strength of the wettability and subsequent adhesion. The strength of the adhesion to retain the nanoparticles is presumably based on intermingling of their organic chains with the sorbent, which cannot be determined directly [38]. Instead, gradual wettability changes of the three-phase system were studied in preliminary tests before their effect was examined during the continuous process. An example of the crude *QDs* synthesis solution, MeOH, and beads with and without treatment is already illustrated in Figure 2. In order to estimate the key players, the list of components was narrowed. For this purpose, neat ODE was combined with three weak solvents (MeOH, EtOH, and ACN), three sorbent surface conditions (hydroxyl, hydrocarbon, or perfluorocarbon groups) and three surfactant types (TOP or OLA or OA). For these surfactants a content of 3 vol% was chosen, according to the phase diagrams as illustrated in Section 3.2.1. A selection of the oppositional cases of ODE with and without OLA are shown in Figure S12. It turns out that SiO_2_ beads modified with hydrocarbon chains guarantee wetting in all factor combinations which becomes visible by the coverage of the solid phase rather than the creation of emulsion droplets. In case of unsilanized beads, using acetonitrile in combination with any surfactant type present in the crude solution leads to the same noticeable capillary forces between the beads and the oil phase. In contrast, methanol delivers a similar picture with unsilanized beads only in combination with oleylamine. When beads with perfluorocarbon chains are used, a Pickering emulsion (oil phase stabilized with beads) can be observed for MeOH in combination with oleylamine or oleic acid, best visible with macroscopic droplets in case of ACN with and without surfactant. Besides, to apparent changes in wetting, the comparison also demonstrates different capacities of the column material. In the case of ethanol, the capacity is lower and a dense distribution of oil drops above the sorbent material is visible, in contrast to methanol and acetonitrile. The same lower capacity becomes clear, comparing beads with different size ranges (250–500 vs. 70–110 μm). Equivalent conclusions in terms of wetting behavior can be drawn from close-up views of plain surfaces from which equilibrium contact angles were obtained, specified in Table 1 and illustrated in Figure S13. Within the ACN series, the maximum contact angle is formed in combination with a perfluorinated surface and decreases from the hydrophilic to hydrophobic sorbent surface condition, even more with OLA. Albeit the lower density of ODE compared to MeOH the sorbent can be covered with the continuous phase when carbon-chains are present at the surface or when the oil is spiked with 3 vol% OLA in case of hydrophilic surface conditions. With regard to the crude synthesis solution (containing ~54 μmol L*^−^*^1^ *QDs*, 19 vol% OLA, 1 vol% OA, and 18 vol% TOP), wetting and extraction happen simultaneously, as can be observed from the dissolving processes in a close-up view (Figure S13). However, the initial contact angles indicate wetting to be strongest with a hydrophobic surface condition or ACN while nanoparticles precipitate with EtOH into agglomerates, which do not adhere on their own.

In the continuous purification scheme dynamic effects are dominant comparable to affinity chromatography, and the separation relies on a balance between sufficient adhesion against shear forces of the fluid medium and prevention of re-adsorption of once-extracted species. This can be realized by carefully taking into account the interplay of weak solvent, sorbent surface condition, and surfactant. For this purpose, the wettability characteristics in equilibrium deliver useful indications. The combination with oleylamine and acetonitrile yields to more wettability, more pronounced than in case of the other two surfactant components and weak solvents. Conversely, in combination with perfluorinated surface conditions, less contact can be realized even with residual surfactant within the continuous phase. With regard to the nanoparticles, being a rather large entity with a molecular layer on the surface, entropy plays a minor role and adhesion forces can be mainly attributed to enthalpic interactions, i.e., intermingling of their surfactant molecules with the organic liquid phase (ODE + OLA) and the organic coating of the column bed.

### 3.2. Continuous Processing

#### 3.2.1. Comparative Factor Analysis

During the method development, the following factors were taken into account in a comparative analysis in a fractional factorial experimental split design (2^4−1^), as illustrated in Table 2 and Table S2. Different types of adhesion and extraction media (protic and aprotic) and their amount in relation to V_Elution_ (V_E_) were considered, as were the sorbent surface condition and the size of sorbent material.

**Figure 4:**
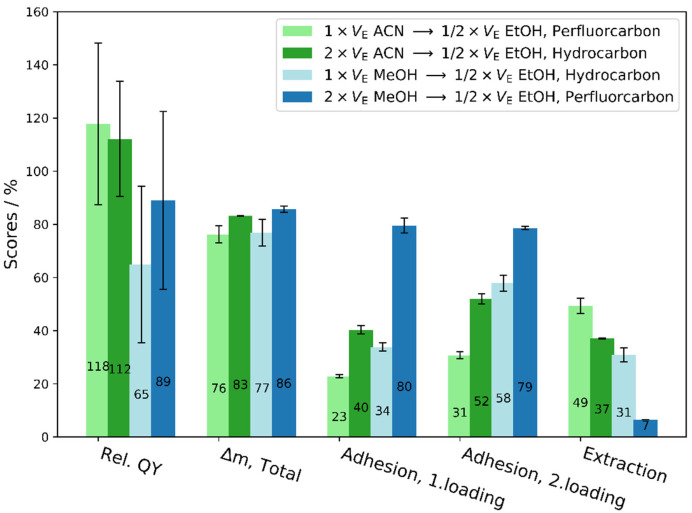
Relative quantum yields (QY) to *QDs* feed solution and separated mass differences (Δm) at different parameter conditions using 70–90 μm SiO_2_-beads. Error bars result from standard deviations running the parameter conditions twice and with respect to the quantum yield from linear regression errors.

**Figure 5:**
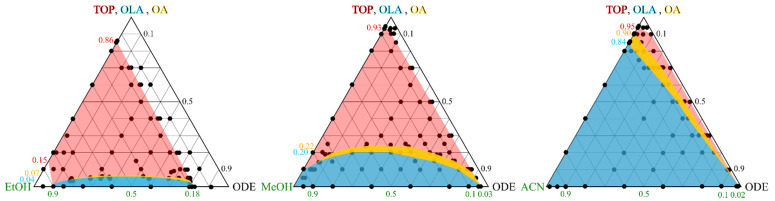
Ternary phase diagrams between original phase (ODE), extraction medium, and synthesis surfactants (TOP, OLA, OA). Dots mark tested volume mixtures, miscibility gaps are color filled.

As response variables, the column capacity was monitored by the relative column height after adhesion of the *QDs*. Impurities which were collected during the adhesion and the subsequent extraction step were analyzed thermogravimetrically to determine the degree of purification. For this, the extracted non-volatile residues were weighted and the ratio to the mass of the original synthesis solvent was taken as the purification score. As an example, purified *QDs* and waste fractions are shown in Figure S6. Although fluorescence can be qualitatively observed in both vessels, the few *QDs* which were not withheld on the column bed and found in the discarded fraction are below the limit of quantification. The limit of quantification (LOQ) is located at 0.007 AU ≈ 0.1 μmol L^−1^ and was determined from the linear regression function shown in Figure S9 after three iteration steps according to L. Sachs [33]. In the same way, concentrations of recovered *QDs* were derived from the first absorption maxima in relation to the initial feed concentration with 54 μmol L^−1^. In this context, a high recovery, regardless of the factor settings, was achieved with 79% in average (see Figure S14). Moreover, the emission intensity in relation to the original quantum yield was used as an additional indicator. The fluorescence of purified *QDs* can be preserved to a high degree even when exposed to ambient atmosphere, which was assessed after a period of two months (See Figure 4). For half of the factor combinations, we observe an increase of the photoluminescence properties, taking slope errors of both linear regression functions, i.e., standard and sample, into account. At the same time, 76–86 mass % could be extracted from the *QDs* stock solution. Both the polarity and the amount of extraction media increase the yield of the purification performance significantly (see ANOVA results Tables S2–S7 and illustrative Figures S15 and S16). At first glance, extraction with twice the elution volume (V_E_) delivers the highest purification yield for methanol compared to acetonitrile. Impurities, which are not eluted by the first medium (methanol or acetonitrile), can be washed off by ethanol, which served as an additional extraction solvent. It was found that the use of ethanol results in an enhanced removal of the impurities relative to the volume invested (1/2 of V_E_). Hence, the sequential combination of methanol and ethanol is most effective for trapping the *QDs* and extracting residual ligands. Consistent with our studies, Woo et al. also reported a higher extraction potential for protic than aprotic solvents [11]. Here, we substantiate these results by ternary phase diagrams shown in Figure 5 between 1-octadecene, three extraction solvents, and the surfactants within the synthesis solution at known volume ratios. From the significantly smaller miscibility gaps with ethanol and OLA or OA compared to methanol or acetonitrile, its higher extraction potential becomes apparent.

With regard to different bead sizes, no statistically significant effect on the purification score of this factor or the combination with other factors was found. In contrast, the influence of the sorbent surface condition (hydrocarbon or perfluorocarbon chains) was confirmed to be significant for the process, as already observed during preliminary experiments (ANOVA results Tables S2–S7). The surface condition of the sorbent contributes as a significant main effect to the purification performance during the first loading of the column bed, being higher with perfluorinated in comparison to hydrocarbon-modified beads. During a second charging step, using the same amount of original synthesis solvent but without prior *QDs* elution, the surface condition is still identified as important, however only in mutual interaction with the amount of solvent. In other words, with less extraction solvent in combination with perfluorinated sorbent material, a higher separation was achieved than with the same amount, but with only hydrocarbon-modified beads independent of the solvent type. The reduced significance originates from coating the beads with adhered *QDs*, yielding to different effective surface properties the sample of the second injection will interact with; nevertheless, multiple loading could be exploited in further optimization to maximize the overall process efficiency. Comparing all significant factors of the ANOVA regression model, the solvent characteristics (protic/aprotic) and their amount applied dominate the linear regression functions which describe the influences upon the oil recovery for the first and second loading step. This can be derived from the amplitude of their coefficients (Equations (S6) and (S7)). As a conclusion, the stock solution can be trapped in a bed with hydrocarbon-modified or perfluorinated beads. The latter shows less pronounced wetting behavior to prevent readsorption of once extracted species, compared to hydrocarbon-modified beads. However, a perfluoroalkyl coating is not necessarily required to achieve a high separation because the gradual difference in adhesion strength is of secondary importance. The extraction medium and its volume invested dominate the overall outcome of the product recovery, for which the sequential usage of methanol and ethanol results in a more effective and efficient purification per volume.

#### 3.2.2. Method Comparison

**Figure 6:**
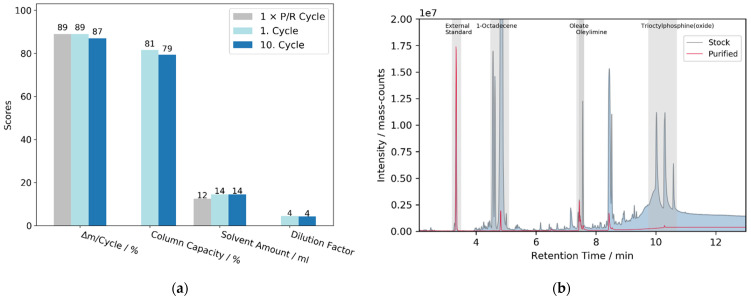
(**a**) Comparison between one time P/R-cycle and multiple SPE cycles using hydrophobic 70–110 μm SiO_2_-beads, methanol as adhesion, and ethanol as extraction medium; (**b**) GC/MS-chromatogram of stock and purified solutions at the same diluting factor.

A direct comparison between the solid-phase extraction approach with a column volume of VC = 24 mL and the classical P/R-method with centrifugation tubes with VT = 50 mL is shown in Figure 6a, both for 2 mL sample volume. Methanol served as the adhesion and ethanol as the extraction solvent on a column bed with 70–90 μm hydrocarbon-modified beads. In total, a comparable result in terms of yield and purification score was achieved with similar amounts of solvents needed. To demonstrate the scalability of the process, multiple cycles were performed, each requiring only around 10 min using flow rates of 10 mL min^−1^ as shown in the chromatogram (Figure S11). In this context, no significant decrease of the column capacity derived from the retention height of the *QDs* or increase of the dilution factor of the original volume was recognized, nor did flow rates show an influence, but they might be raised up even higher to maximize the overall process efficiency.

To evaluate the purification outcome in more detail, GC/MS-analysis was performed of supernatants which were obtained after thorough sedimentation of agglomerated nanoparticles via centrifugation. A direct comparison with same dilution factors shows a major decrease of impurities between stock and purified samples (see Figure 6b). The signal at a residence time of 8.4 min is a reaction side product of zinc diethyldithiocarbamate according to the mass spectrum. Oleic acid could be removed to below the detection limit within the purified *QDs* solution. The residual concentration of 1-octadecene, oleylamine, and trioctylphosphine are 1%, 1%, and 3% with respect to the original amount detected within the stock solution by means of external calibration measurements (see Figure S10).

#### 3.2.3. Hansen Solubility Parameter of QDs

The origin of the Hansen solubility sphere of hydrophobic *QDs* was determined to be at (δD = 16.9 MPa^1/2^, δP = 1.6 MPa^1/2^, δH = 3.9 MPa^1/2^) with a small radius of 5.8 MPa^1/2^ using the experimental solvent classification data listed in Table S1. The sphere volume and its origin was modeled in accordance with the HSP sphere technique [20]. The non-linear evolutionary algorithm integrated within Microsoft Excel was used, resulting in a fit score of 92% (Total amount of test solvents = 52 (15 good, 37 bad), Wrong out = 0, Wrong in = 3) [39]. Another elaborated modeling strategy has been proposed recently, applying saturation concentrations of the target in order to account for the gradual goodness of solvents as considered theoretically [40]. This modeling technique was not applied here, since no significant differences were recognized experimentally throughout the HSP space.

**Figure 7:**
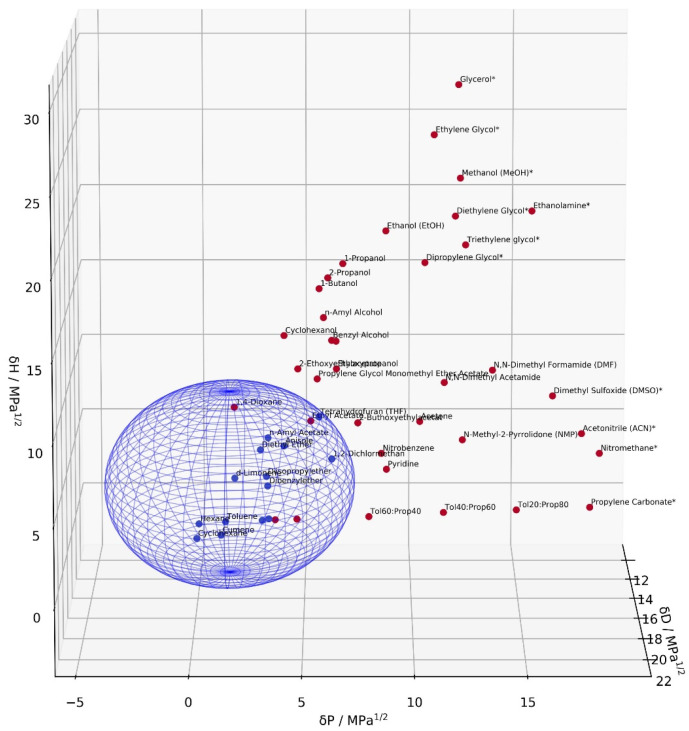
Solubility property 1 mg CdSe/ZnS *QDs* within the HSP space. The blue sphere indicates the predicted compatibility area. Red dots indicate poor compatibility. In this context, stars indicate a miscible behavior with the stock synthesis solution, hence adhesion onto the column bed.

Figure 7 shows the obtained HSP sphere. This HSP overview provides new candidates for the purification procedure. Compatible solvents are shown in blue, whereas the non-compatibility behavior is marked in red. In this context, non-miscibility of the stock solution without nanoparticle precipitation is highlighted via a star. With latter solvents, adhesion of the *QDs* to the sorbent material was expected and experimentally confirmed. Within the blue sphere, new compatibility areas are disclosed. From this range, new solvent blends can be selected to successfully elute and subsequently store the *QDs* while maintaining colloidal stability. In addition, a rational choice upon integration of purified *QDs* within new matrices, e.g., polymers, can be made. This is consistent with an encapsulation study of phosphonic- and phosphine oxide-stabilized CdSe *Q*Ds in a biphenylperfluorocyclobutyl polymer in contrast to siloxanes and epoxy resins [41]. In the latter study, the HSP vector distances of the tested substances were in fact estimated. However, the overall trend comparing different classes of the HSP study could be ascertained. Here, the increased δH = 3.9 value of *QDs* compared to ODE [16.1, 1.2, δH = 1.8] indicates that the solubility of nanoparticles is more determined by the hydrogen bonding property of its surfactants. This is in accordance with their higher affinity to the column bed observed during the purification procedure when oleylamine is included, for which the HSP value is not known yet, but can be estimated from the homologous series (propylamine [16.9, 4.9, 8.6] and butylamine [16.2, 4.5, 8.0]).

**Figure 8:**
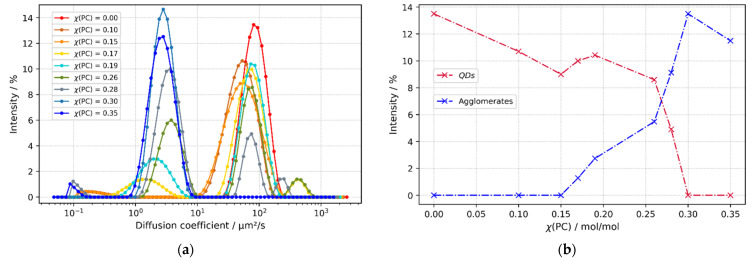
Comparison of diffusion coefficient distributions of different Toluene-*QDs* solutions with increasing molar propylene carbonate content χ(PC) derived from dynamic light scattering experiments. (**a**) Scattering intensity over a range of diffusion coefficients. (**b**) Maximum intensity of both scattering modes over a range of propylene carbonate molar content χ(PC).

The starting point of the agglomeration can be identified distinctively from dynamic light scattering experiments, looking at the diffusion coefficient, as shown in Figure 8a. In this context, a scattering mode with a diffusion coefficient of 69 μm^2^/s in average can be assigned to fully dispersed *QDs*, while the second mode with a maximum at 2–4 μm^2^ s^−1^ can be assigned to initial agglomerated particles. As expected, the maximum signal intensity of the latter progressively increases while the fully dispersed *QDs* mode decreases when more propylene carbonate (PC) is added, as shown in Figure 8b. The beginning of the flocculation is located between a PC molar ratio (χ) of 0.15 and 0.17, when the second mode appears. Additionally, it is possible to determine the hydrodynamic radii of both groups due to known viscosities of the solvent blend, measured by G. Ritzoulis et al. [36]. In the case of fully dispersed *QDs*, a hydrodynamic radius R_h_ = 3–6 nm is in good agreement with radii obtained from TEM images, as shown in Figure S17. Meanwhile, the R_h_ of the initial agglomerates is found to be significantly larger, 170 nm in average.

The complexity of nanoparticles surfactant interdependence with solvents cannot be explained thoroughly via HSP, as shown in the past for a homologous series [23]. However, this assessment is inexpensive and convenient to map an experimental overview of the intermolecular energy contributions of a new chemical system, contributing to the database in general. Here, we determined the compatibility area of 1 mg CdSe/ZnS *QDs* with short-chained (OLA, OA, TOP), common “hydrophobic” surfactants at their particle surface. For this purpose, the DLS principle delivers the transition point more objectively than visual experiments alone because the appearance of even small particle agglomerates causes a distinct scattering signal. Due to the widespread availability of DLS instruments, it serves as a sensitive measurement approach for a HSP study with regard to very small sized nanoparticles.

## 4. Conclusions

This work demonstrates how purification of common oil-based CdSe/ZnS *QDs* synthesis solutions can be automated and accelerated without prior sample treatments by the use of a solid phase extraction scheme and corresponding technical setup. Sufficient adhesion of *QDs* is created by silane-covered nonporous SiO_2_ beads. This low-cost stationary phase material allows scalability of the loading capacity and fast elution of purified nanoparticles solutions, minimizing manual handling. Systematic parameter studies reveal that the amount and characteristics of the extraction solvents are the key parameters for retention of the *QDs* and the purification yield (comparing acetonitrile, methanol, and ethanol). An effective exchange of the original solvent/ligand matrix is achieved comparable to one conventional precipitation/centrifugation step. A high reduction of the excess ligand molecule concentration was observed via GC/MS-analysis. Towards an efficient and cost-effective preparative purification technique of *QDs* in organic solvents, this study contributes towards fast implementation by transitioning from batch to flow-processing. As the purification scheme is based on the molecular interactions of the *QDs* whose properties were determined within a Hansen solubility parameter analysis, column material, and solvents, the concept can be expected to work as well with other hydrophobic nanomaterials.

## Figures and Tables

**Table 1:** Equilibrium contact angles within the ternary phase system, i.e., sorbent, sample solution, and extraction medium.

Sorbent	Hydrophilic		Hydrophobic		Perfluorinated	
Solvent	ODE	ODE + OLA	ODE	ODE + OLA	ODE	ODE + OLA
ACN	71°	21°	45°	22°	91°	58°
MeOH	No contact	73°	61°	47°	90°	109°
EtOH	No contact	70°	65°	58°	No contact	No contact

**Table 2:** Specification of factor conditions within a comparative ANOVA study.

Solvent	Amount	Sorbent	Ø/μm
MeOH	1 × V_E_	Hydrocarbon	70–110
ACN	2 × V_E_	Perfluorocarbon	250–500

## Data Availability

The data presented in this study are available on request from the corresponding author.

## Supplementary Materials

The following are available online at https://www.mdpi.com/article/10.3390/nano12121983/s1, Figure S1: Flow-charts of continuous synthesis and purification of CdSeZns *QDs*, Figure S2: Interdigital and caterpillar micro mixer developed and manufactured at Fraunhofer IMM, Mainz, Germany, Figure S3: Combined absorbance and emission spectrum of continuously synthesized green CdSe/ZnS *QDs* used for purification studies, Figure S4: Thermogravimetric analysis of *QDs* stock solution indicating three mass loss steps at 240, 323 and 422 °C, Figure S5: SiO_2_ beads before and after silanization with OTMS and PFOCTS, Figure S6: Collecting vessels from one purification cycle under UV-light after heat treatment, Figure S7: Column case, fittings and frit used for the stationary phase, Figure S8: Residence time distribution within the column bed from fluorescence emission signal of Rhodamine 6G as tracer material, Figure S9: Quantum yield analysis from integrated fluorescence signals of an exemplary sample of purified *QDs* and Rh6G as standard, Figure S10: GS/MS Results and calibration curves from repetitive measurements of the surfactants, stock and purified *QDs* solutions at different diluting factors, Figure S11: In-line absorbance signal at 360 nm and fluorescence signal at 534 nm during one purification cycle, Figure S12: Juxtaposition of different adhesion behavior of non-treated and treated beads, colored 1-octadecene with and without OLA, Figure S13: Juxtaposition of gradual wettability changes via equilibrium contact angles in the three phase system containing extraction medium (ACN, MeOH, EtOH), ODE with and without 3 vol% OLA or *QDs*-synthesis solution, Figure S14: Elution volume and residual *QDs* concentration after the purification scheme in relation to the stock concentration, Figures S15 and S16: Predicted mass loss from regression model functions versus actual mass loss used for ANOVA analysis for the first and second loading step, Figure S17: Distribution of Stokes radii of *QDs* in toluene derived from light scattering experiments from five individual measurements and TEM image of the corresponding *QDs* sample, Table S1: Solvent list of the Hansen Solubility Parameter Study and scoring of their compatibility, Table S2: Experimental plan with one repetition of the 2^(4−1)^ fractional factorial split-design, Tables S3–S7: ANOVA result tables and fit statistics for the response variable “mass loss” for the first and second loading step.
