# Supplementary material for: Automated Quantum Dots Purification via Solid Phase Extraction
Source: Nanomaterials (Basel). 2022 Jun 9;12(12):1983. doi: 10.3390/nano12121983 (PMC9230973; doi:10.3390/nano12121983)
Supplement: Supplementary file 1 [file nanomaterials-12-01983-s001.zip › nanomaterials-1713582-supplementary materials.pdf]

nanomaterials

Supplementary Materials

Automated *Quantum Dots* Purification via Solid Phase Extraction

Malín G. Lüdicke [1,*], Jana Hildebrandt [1,2], Christoph Schindler [1,3], Ralph A. Sperling [1,*] and Michael Maskos [1]

[1] Fraunhofer Institute for Microengineering and Microsystems IMM, 55129 Mainz, Germany; jana.hildebrandt@bam.de (J.H.); c.schindler@interbran.de (C.S.); michael.maskos@imm.fraunhofer.de (M.M.)
[2] Federal Institute for Materials Research and Testing, 12205 Berlin, Germany
[3] Interbran Advanced Materials GmbH, 76684 Oestringen, Germany
* Correspondence: malin.luedicke@imm.fraunhofer.de (M.G.L.); ralph.sperling@imm.fraunhofer.de (R.A.S.)

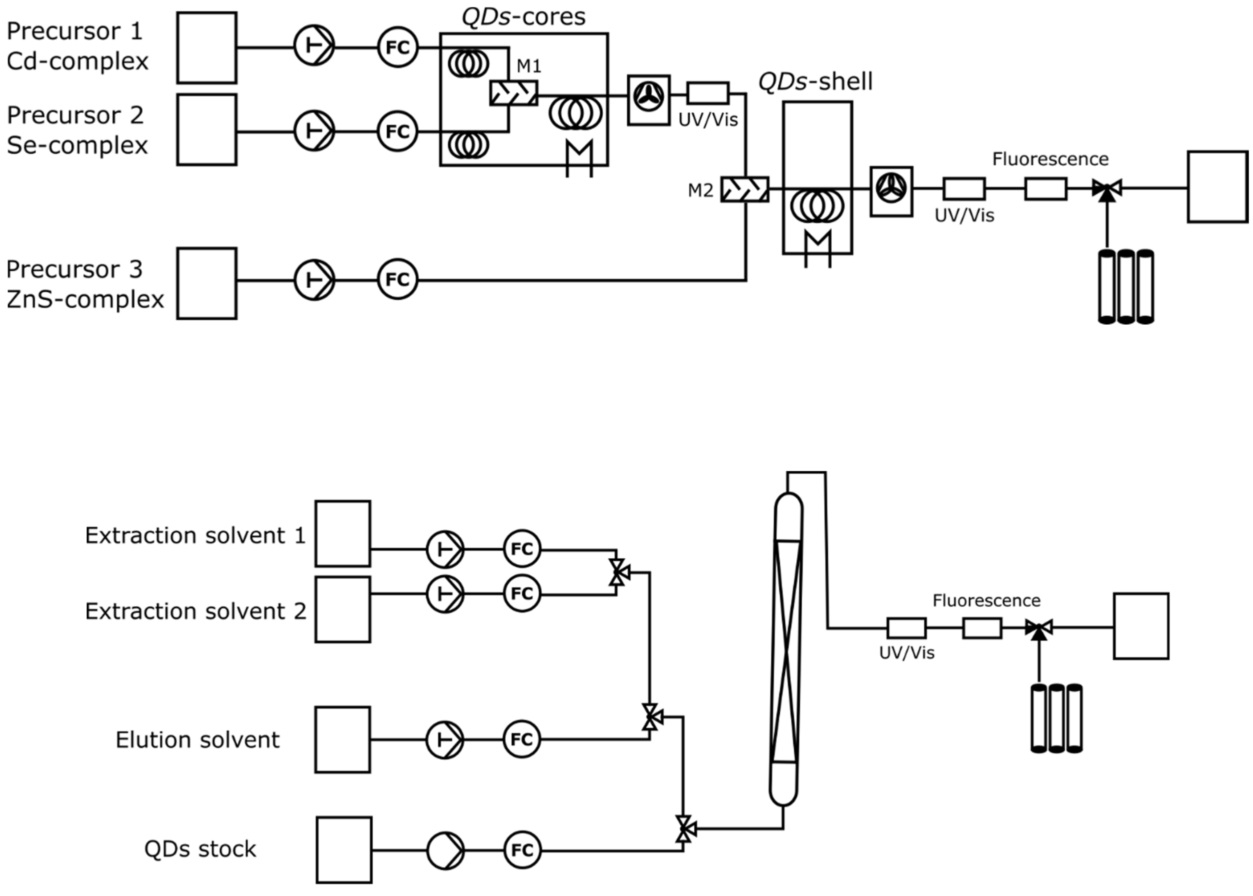

Figure S1. Flow-charts of continuous synthesis (top) and purification (bottom) of CdSeZns *QDs*.

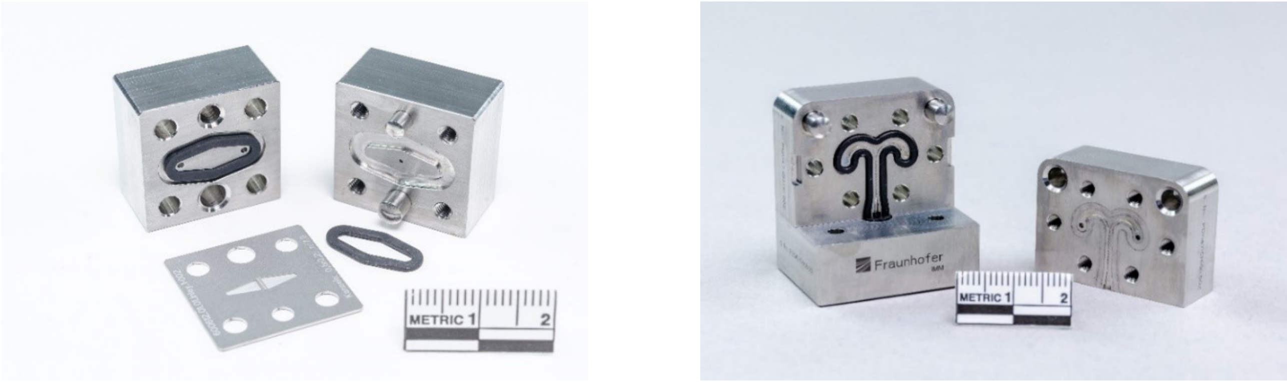

(**a**) (**b**)

Figure S2. (**a**) Interdigital (**b**) caterpillar micro mixer developed and manufactured at Fraunhofer IMM, Mainz, Germany**.**

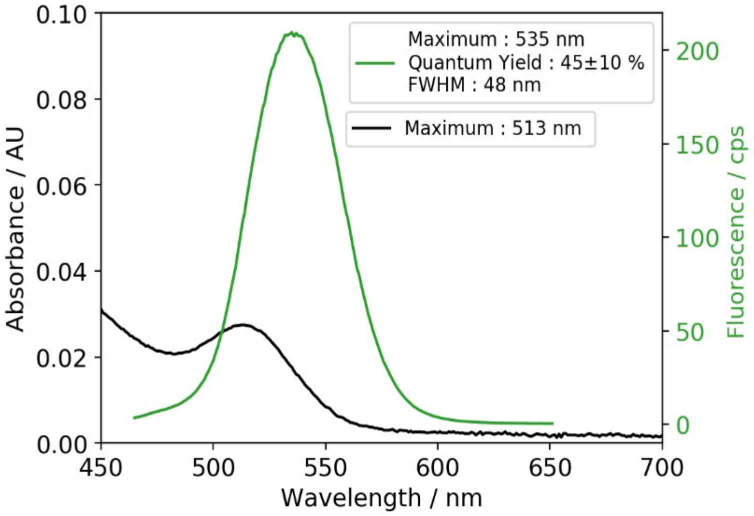

Figure S3. Combined absorbance and emission spectrum of continuously synthesized green CdSe/ZnS *QDs* used for purification studies.

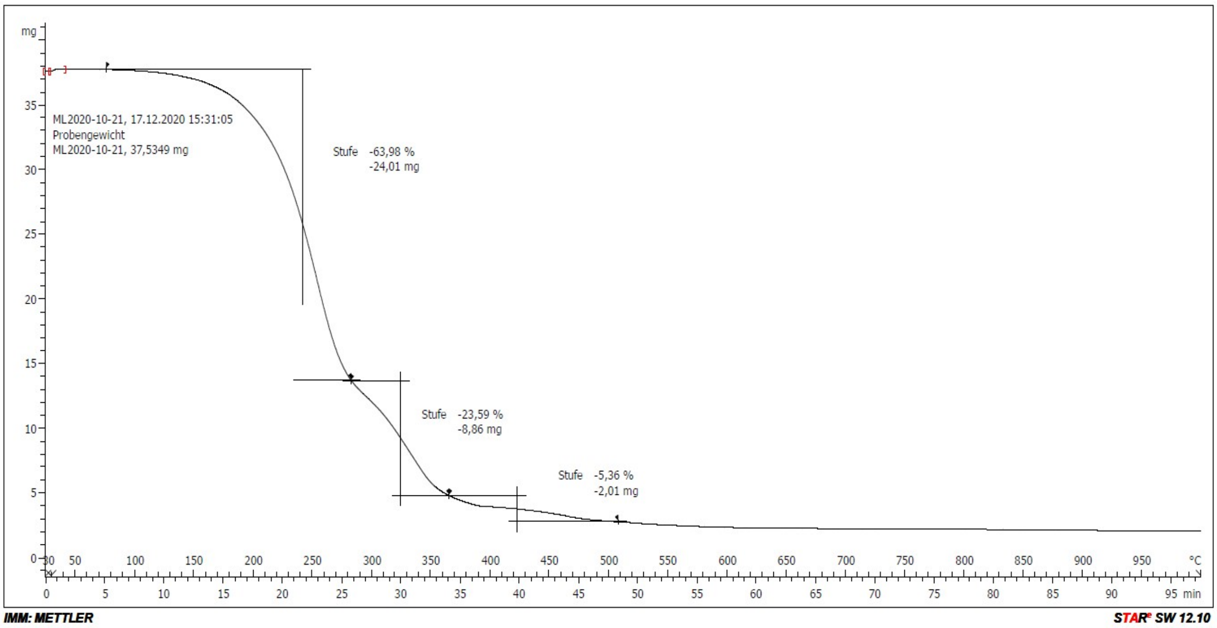

Figure S4. Thermogravimetric analysis of *QDs* stock solution indicating three mass loss steps at 240, 323 and 422 °C.

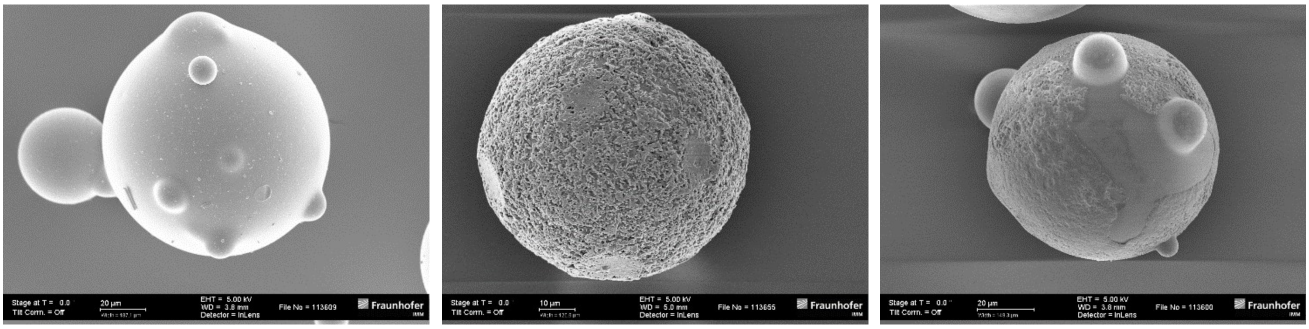

Figure S5. SiO_2 beads before (left) and after silanization with OTMS (middle) and PFOCTS (right).

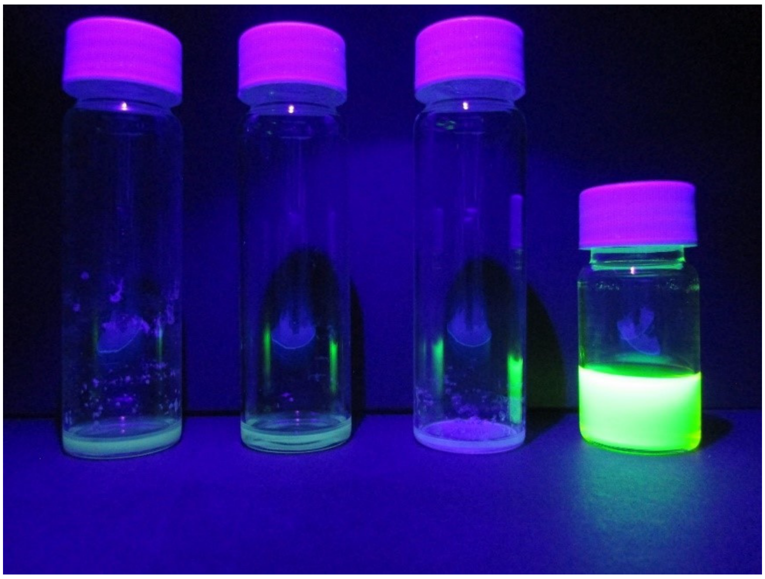

Figure S6. Collecting vessels from one purification cycle under UV-light after heat treatment. Non-volatile synthesis components collected during the adhesion process (1st and 2nd loading) (left) and extraction process (middle) process step. (Right) *QDs* solution collected after purification with a diluting factor of 4 in relation to the original volume.

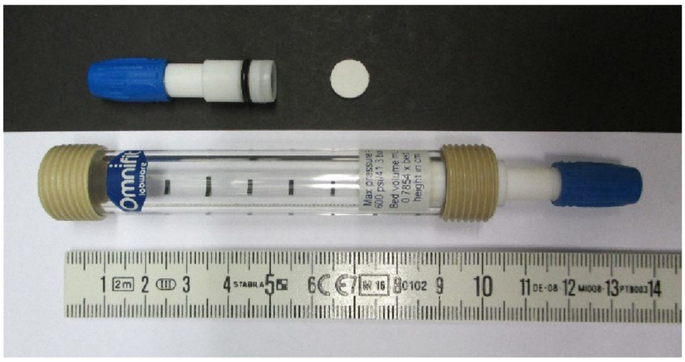

Figure S7. Column case, fittings and frit used for the stationary phase.

$$E(t) = \frac{I(t)}{\int_0^{\infty} I(t)dt} \approx \frac{I(t)}{I_{total}} = \frac{I(t)}{\sum_0^n I(t_i)} \quad (1)$$

$$F(t) = \int_0^{\infty} E(t)dt \approx \sum_0^n E(t_i) \quad (2)$$

$$t_m = \int_0^{\infty} t \cdot E(t)dt \quad (3)$$

$$\sigma^2 = \int_0^{\infty} t^2 \cdot E(t)dt - {t_m}^2 \quad (4)$$

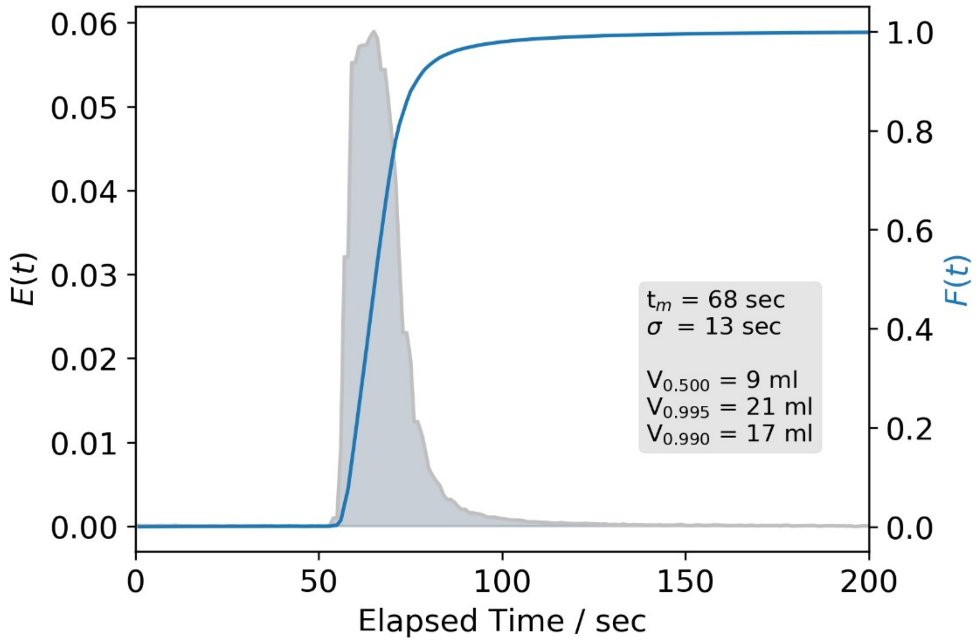

Figure S8. Residence time distribution within the column bed from fluorescence emission signal of Rhodamine 6G as tracer material.

$$\frac{QY}{\%} = QY_{max.,standard} \cdot \frac{b_{sample} \cdot n^2_{sample}}{b_{standard} \cdot n^2_{standard}} \tag{5}$$

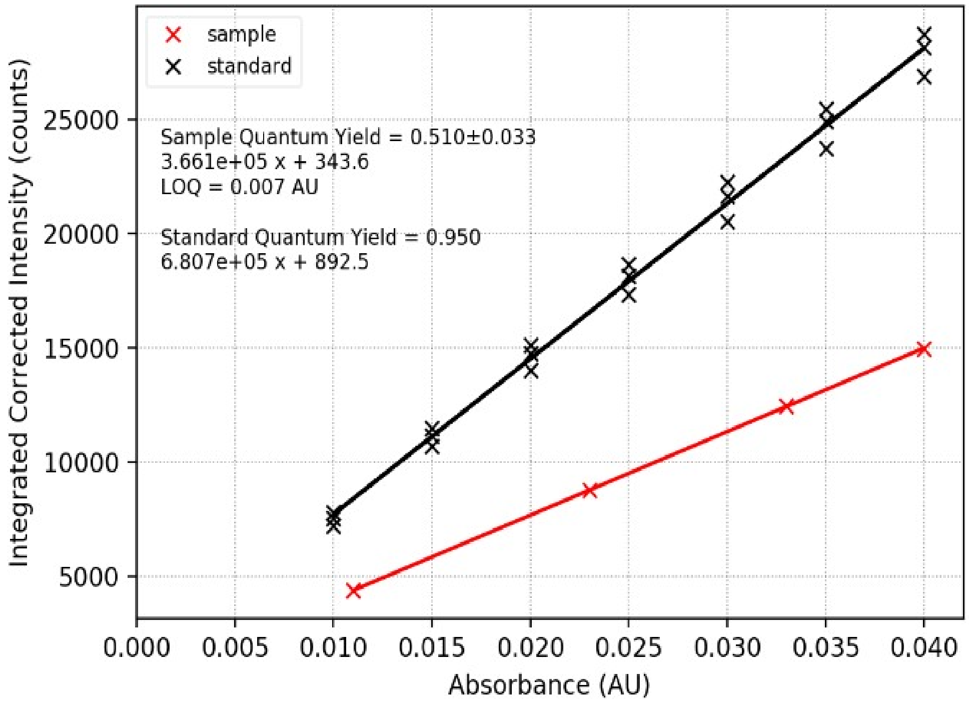

Figure S9. Quantum yield analysis from integrated fluorescence signals of an exemplary sample of purified *QDs* and Rh6G as standard.

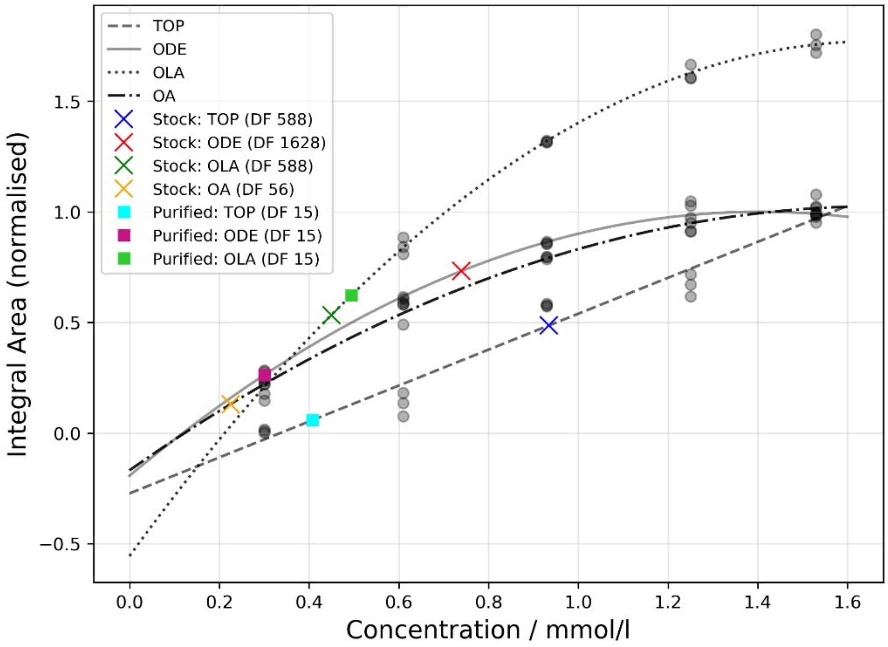

Figure S10. GS/MS Results and calibration curves from three repetitive measurements of trioctylphosphine (TOP), 1-octadecene (ODE), oleylimine (OLA) and oleate (OA), and two repetitive measurements of stock and purified *QDs* solutions at different diluting factors (DF). Gray markers: Calibration standards, colored markers: Samples.

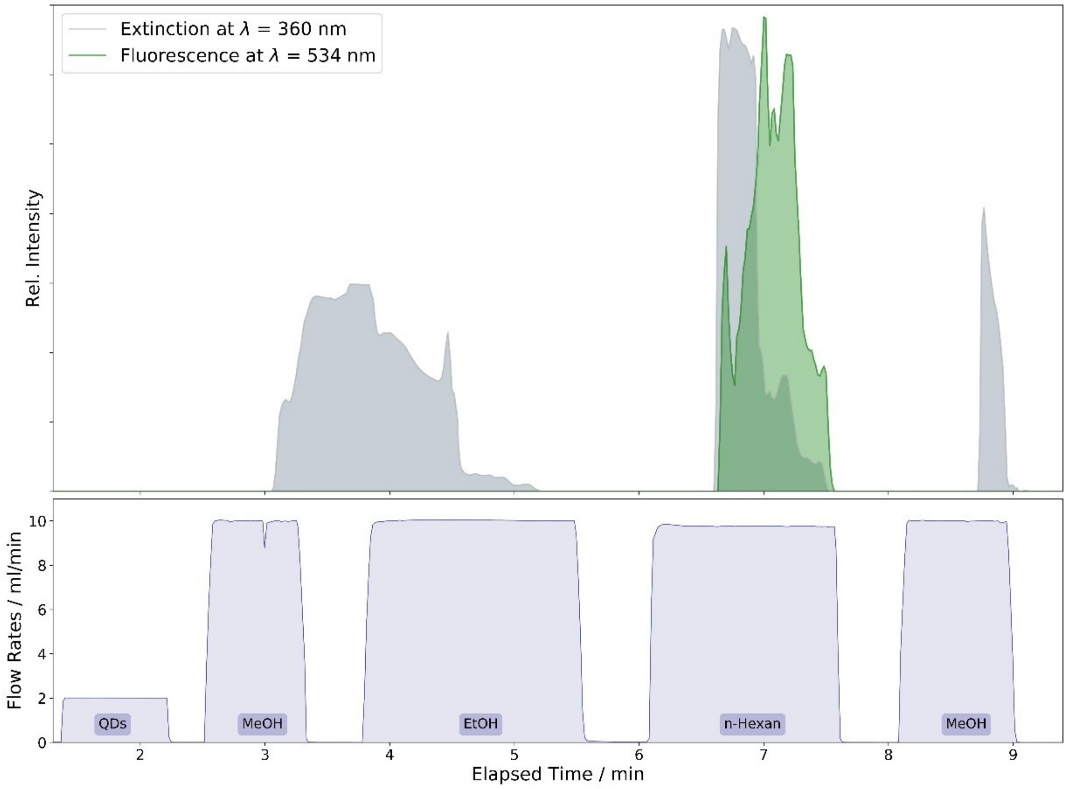

Figure S11. In-line absorbance signal at 360 nm and fluorescence signal at 534 nm during one purification cycle including loading of 1.) *QDs*, 2.) adhesion with methanol, 3.) extraction with ethanol, 4.) elution with n-hexane and 5.) pre-conditioning with methanol with flow rates of 2 and 10 ml/min respectively.

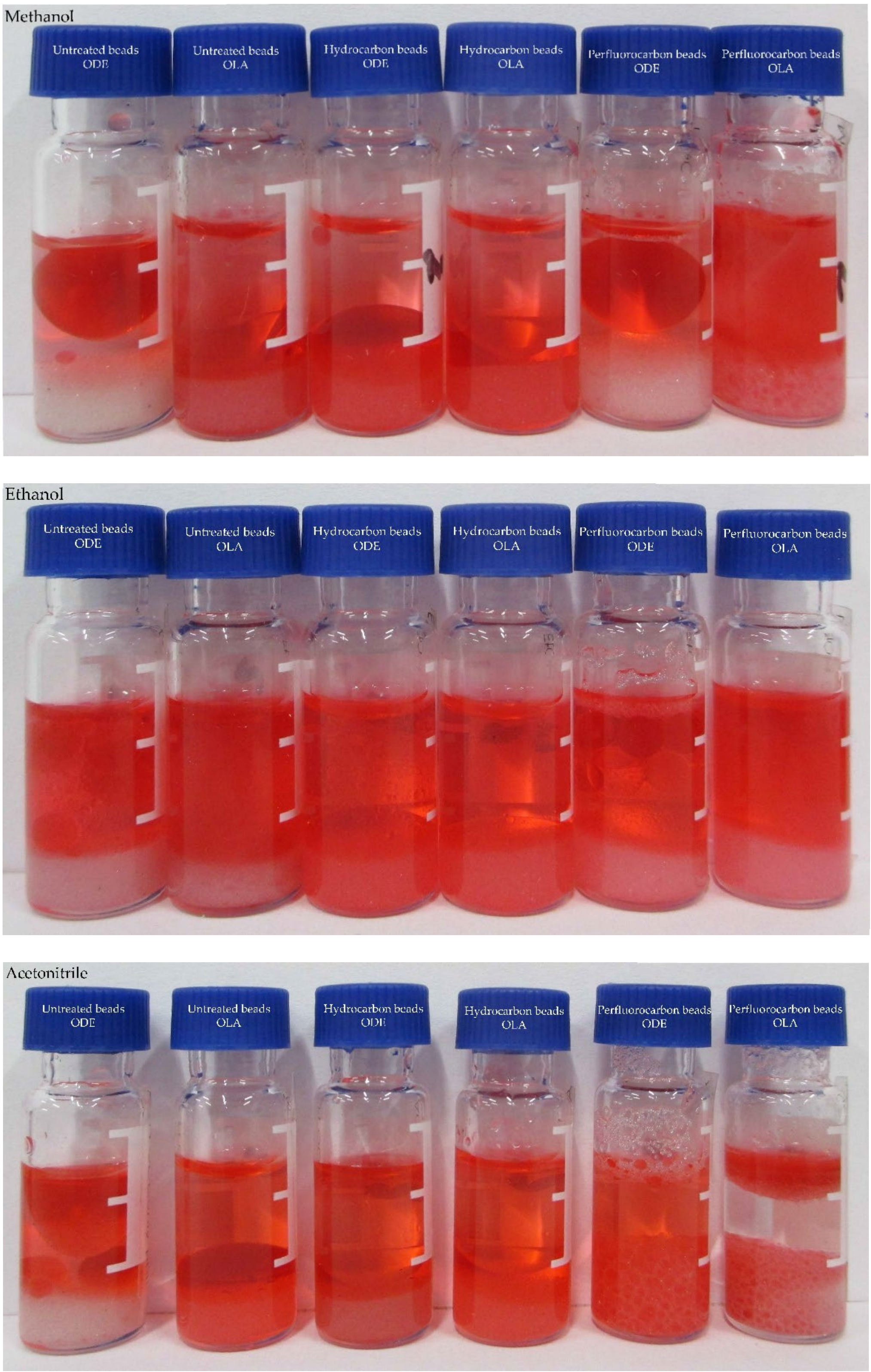

Figure S12. Juxtaposition of different adhesion behavior of non-treated and treated beads and colored 1-octadecene (Oil-Red-O Sigma Aldrich) with and without 3 vol % of surfactant OLA from top to down: methanol, ethanol, acetonitrile.

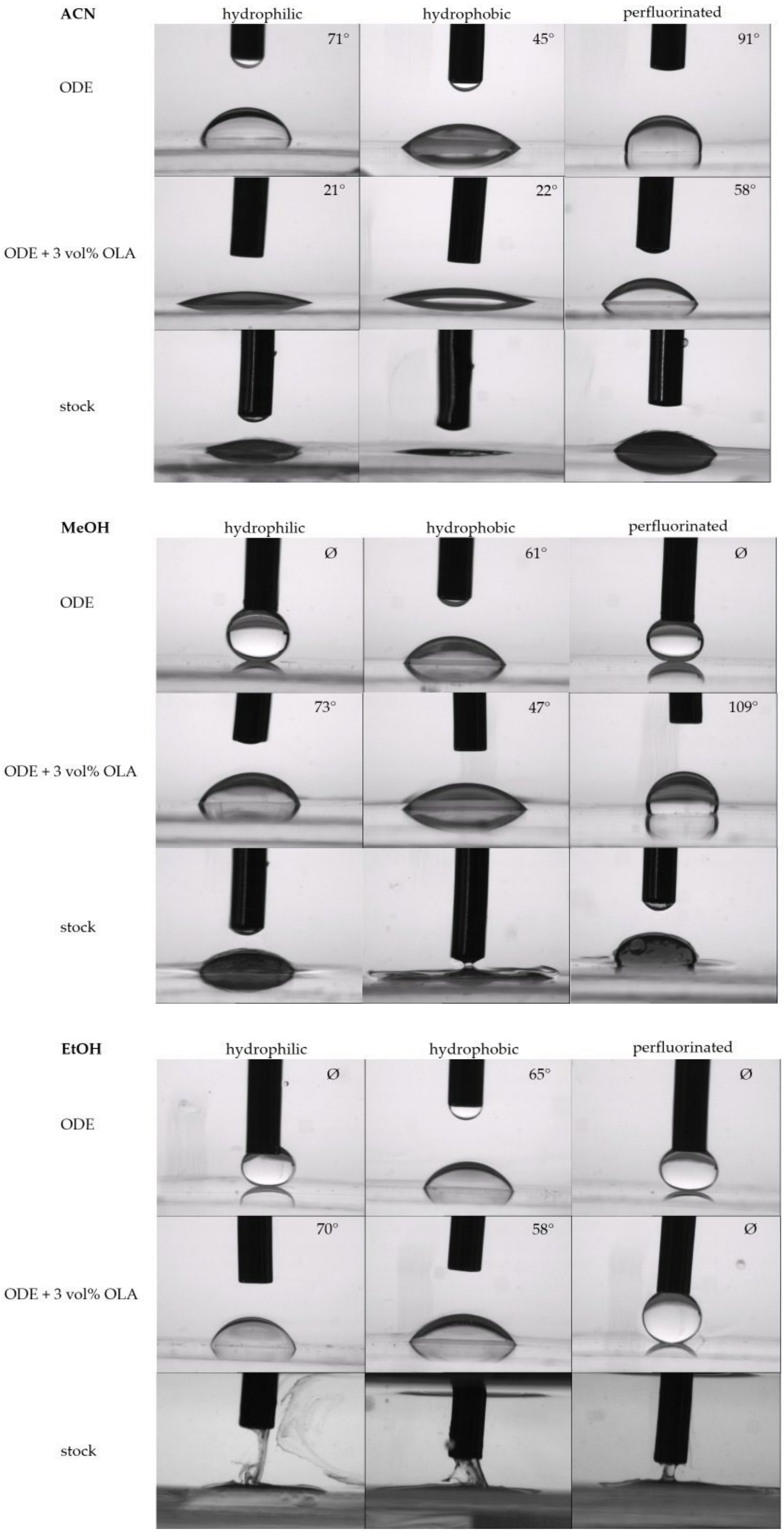

Figure S13. Juxtaposition of gradual wettability changes via equilibrium contact angles in the three phase system containing extraction medium (ACN, MeOH, EtOH), ODE with and without 3 vol% OLA or *QDs*-synthesis solution.

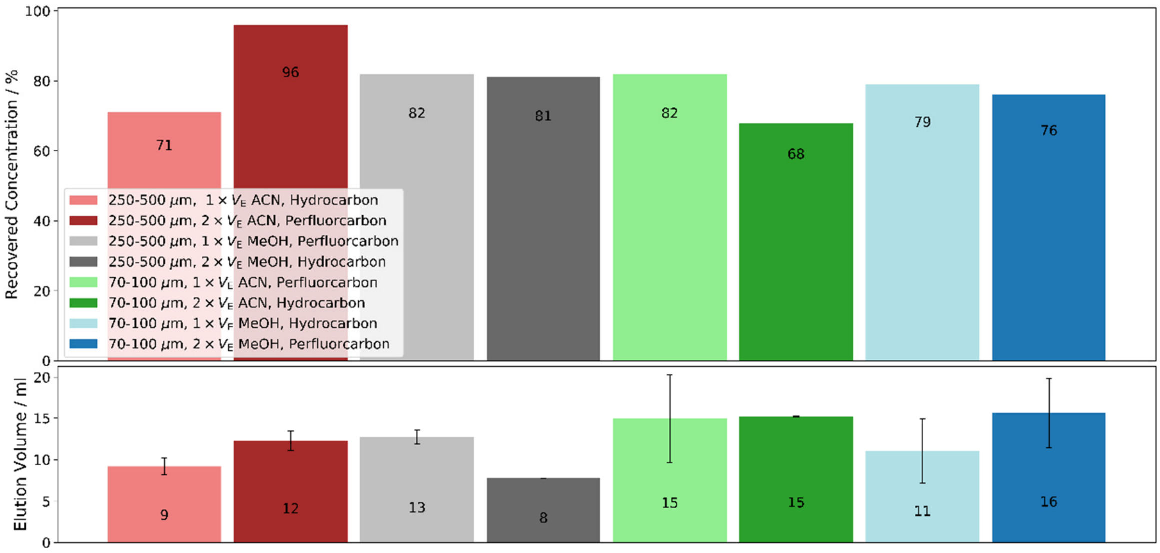

Figure S14. Elution volume and residual *QDs* concentration after the purification scheme in relation to the stock concentration. In this context all parameter setting combinations including size of sorbent material, amount (multitude of elution volume (V_E)) and type of precipitation medium and sorbent surface conditions are displayed.

Table S1. Solvent list of the Hansen Solubility Parameter Study and scoring of their compatibility. (1) means compatibility and (0) the opposite. (WI) wrong-in and (WO) wrong-out are error indicators and refer to solvents and non-solvents respectively whose experimental compatibility behavior is opposed to the modeling result.

Chemical	Purity	Supplier	Scoring	WI/WO
Acetone	100	VWR Chemicals	0	-
Acetonitrile	99.8	Sigma Aldrich	0	-
n-Amyl Acetate	98	Merck KGaA	0	-
n-Amyl Alcohol	99	Sigma Aldrich	0	-
Anisol	99	Fluka	0	-
Benzyl Alcohol	99	Fluka	0	
1-Butanol	99.5	PanReac AppliChem	0	-
2-Buthoxyethylacetat		Polymer Merck	0	-
m-Cresol	98	Fluka	0	-
Chloroform	100	VWR Chemicals	1	
Cyclohexane	99.5	VWR Chemicals	1	-
Cyclohexanol	98.5	Roth	0	-
Cumene	98	Aldrich Chemical Company	1	-
Dibenzylether	98	Alfa Aesar	1	-
1,2-Dichlormethane	99.8	CHEMSOLUTE	1	-
Diethylether	99.5	VWR Chemicals	1	-
Diethylene Glycol	99.9	Sigma Aldrich	0	-
Diisopropylether	99	Merck	1	
Dimethyl Sulfoxide	99.5	Merck KGaA	0	-
1,4-Dioxane anhydrous	99.8	Sigma Aldrich	1	WI
Dipropylene Glycol	99	Alfa Aesar	0	-
d-Limonene	97	Alfa Aesar	1	-
Ethanol absolute	99.5	VWR Chemicals	0	-
Ethyl Acetate	99.8	VWR Chemicals	0	WI
Ethylene Glycol	99	Sigma Aldrich	0	-
Ethanolamine	99.5	Merck KGaA	0	-
2-Ethoxyethylacetate	98	Alfa Aesar	0	-
Ethoxy-2-Propanol	95	ACROS Organics	0	-
Glycerol anhydrous	99,5	Merck	0	-
Heptane	99.8	VWR Chemicals	1	-
Hexane	99	Carl Roth	1	-
Methanol	99.9	Carl Roth	0	-
Methyl Ethyl Ketone	99.5	Riedel-de Haën	0	-
N-Methyl-2-Pyrrolidone	98	Fluka	0	-
N,N-Dimethyl Acetamide	99.9	Scharlau	0	-
N,N-Dimethyl Formamide	99.9	VWR Chemicals	0	-
Nitrobenzene	99	Sigma Aldrich	0	-
Nitromethan	98.5	Carl Roth	0	-
2-Propanol	99.9	CHEMSOLUTE	0	-
1-Propanol	99.5	CHEMSOLUTE	0	-
Propylene Carbonate anhydrous	99.7	Sigma Aldirch	0	-
Propylene Glycol Monomethyl Ether Acetate	99	Fluka	0	-
Pyridine	99	Sigma Aldrich	0	-

Tetrahydrofuran	99.9	CHEMSOLUTE	1	-
Triethylene glycol	99	Sigma Aldrich	0	-
Toluene anhydrous	99.8	Sigma Aldrich	1	-
Tol20:Prop80	99.8/99.7	Sigma Aldrich	0	-
Tol40:Prop60	99.8/99.7	Sigma Aldrich	0	-
Tol60:Prop40	99.8/99.7	Sigma Aldrich	0	-
Tol80:Prop20	99.8/99.7	Sigma Aldrich	0	WI
Tol86:Prop14	99.8/99.7	Sigma Aldrich	0	WI
Tol88:Prop12	99.8/99.7	Sigma Aldrich	1	-
Tol90:Prop10	99.8/99.7	Sigma Aldrich	1	-

Analysis of variance (ANOVA)

Table S2. Experimental plan with one repetition of the factor conditions ($2^{(4-1)}$ fractional factorial split design). Factor 1: Surface condition of sorbent, factor 2: SiO_2 bead size in µm, factor 3: Adhesion medium methanol (MeOH) or acetonitrile (ACN) and factor 4: Amount of extraction solvent relative to effective elution volume (V_E) along the flow path.

Run-order	Group	Factor 1	Factor 2	Factor 3	Factor 4
1	1	Perfluorocarbon	70-110	MeOH	2* $V_{Elution}$
2	1	Perfluorocarbon	70-110	ACN	1* $V_{Elution}$
3	2	Hydrocarbon	70-110	MeOH	1* $V_{Elution}$
4	2	Hydrocarbon	70-110	ACN	2* $V_{Elution}$
5	3	Hydrocarbon	70-110	ACN	2* $V_{Elution}$
6	3	Hydrocarbon	70-110	MeOH	1* $V_{Elution}$
7	4	Perfluorocarbon	250-500	MeOH	1* $V_{Elution}$
8	4	Perfluorocarbon	250-500	ACN	2* $V_{Elution}$
9	5	Hydrocarbon	250-500	ACN	1* $V_{Elution}$
10	5	Hydrocarbon	250-500	MeOH	2* $V_{Elution}$
11	6	Perfluorocarbon	70-110	ACN	1* $V_{Elution}$
12	6	Perfluorocarbon	70-110	MeOH	2* $V_{Elution}$
13	7	Perfluorocarbon	250-500	MeOH	1* $V_{Elution}$
14	7	Perfluorocarbon	250-500	ACN	2* $V_{Elution}$
15	8	Hydrocarbon	250-500	ACN	1* $V_{Elution}$
16	8	Hydrocarbon	250-500	MeOH	2* $V_{Elution}$

Table S3. ANOVA result table (REstricted Maximum Likelihood-Kenward-Roger p-values) for the response variable "mass loss" for the first loading step with respect to the factor 1,2,3,4 and their mutual interaction.

Source	Term df	Error df	F-value	p-value	Significance
**Whole-Group**	3	10.0	3.76	0.0484	significant
a-Sorbent Surface Condition	1	10.0	8.24	0.0167	significant
b-Sorbent Size	1	10.0	1.36	0.2713	not significant
ab	1	10.0	2.11	0.1770	not significant
**Sub-Group**	2	10.0	32.51	< 0.0001	significant
C-Extraction Solvent Type	1	10.0	20.33	0.0011	significant
D-Extraction Solvent Volume	1	10.0	27.42	0.0004	significant

P-values less than 0.0500 indicate model terms are significant. In this case a, C, D are significant model terms. Values greater than 0.1000 indicate the model terms are not significant.

Table S4. Fit statistics for the model used for ANOVA with respect to the experimental data of the first loading step.

**Std. Dev.**	8.90	**R^2**	0.8752
**Mean**	44.06	**Adjusted R^2**	0.7659
**C.V. %**	20.20		

Model equation variables according to table above

$$\text{mass loss} = 44.1 + 6.6 \cdot a + (-2.7) \cdot b + 11.1 \cdot C + 12.1 \cdot D + 3.2 \cdot ab \quad (6)$$

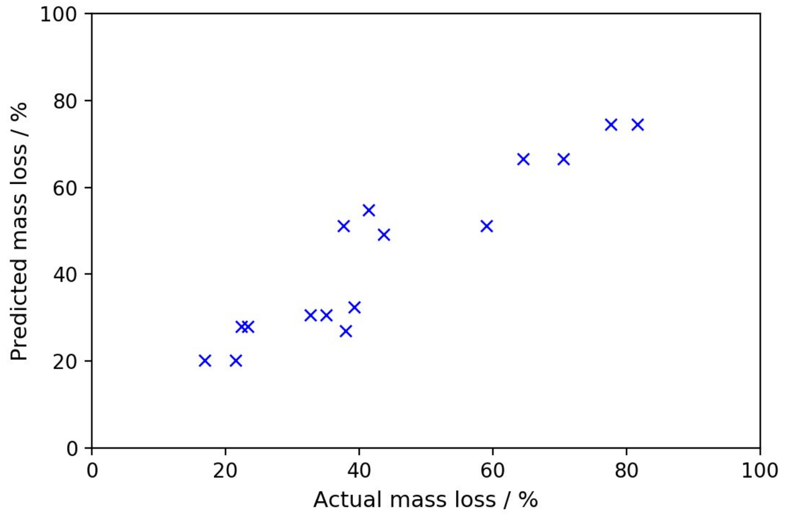

Figure S15. Predicted mass loss from the regression model function versus actual mass loss used for ANOVA analysis for the first loading step.

Table S6. ANOVA result table (REstricted Maximum Likelihood-Kenward-Roger p-values) for the response variable "mass loss" for the second loading step with respect to the factor 1,2,3,4 and their mutual interaction.

Source	Term df	Error df	F-value	p-value	Significance
**Whole-Group**	3	4.00	0.0580	0.9792	not significant
a-Sorbent Surface Condition	1	4.00	0.0635	0.8135	not significant
b-Sorbent Size	1	4.00	0.1077	0.7592	not significant
ab	1	4.00	0.0029	0.9599	not significant
**Sub-Group**	4	4.00	572.40	< 0.0001	significant
C-Extraction Solvent Type	1	4.00	1618.69	< 0.0001	significant
D-Extraction Solvent Volume	1	4.00	656.90	< 0.0001	significant
aC	1	4.00	1.47	0.2915	not significant
aD	1	4.00	12.55	0.0240	significant

P-values less than 0.0500 indicate model terms are significant. In this case C, D, aD are significant model terms.

Table S7. Fit statistics for the model used for ANOVA with respect to the experimental data of the second loading step.

**Std. Dev.**	**2.55**	**R^2**	**0.9952**
**Mean**	54.14	**Adjusted R^2**	0.9879
**C.V. %**	4.71		

Model equation variables according to table above

$$\text{mass loss} = 54.1 + 0.2 \cdot a + 0.3 \cdot b + 15.1 \cdot C + 9.6 \cdot D + (-0.04) \cdot ab + (0.5) \cdot aC + (-1.3) \cdot aD \quad (7)$$

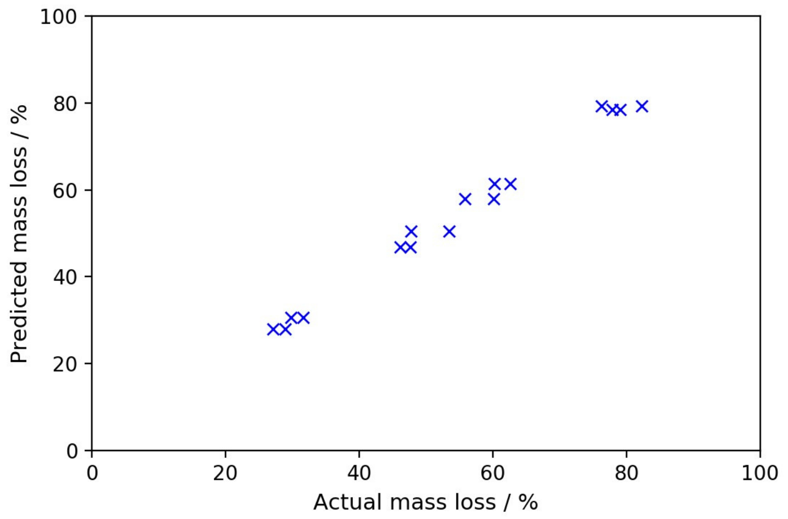

Figure S16. Predicted mass loss from the regression model function versus actual mass loss used for ANOVA analysis for the second loading step.

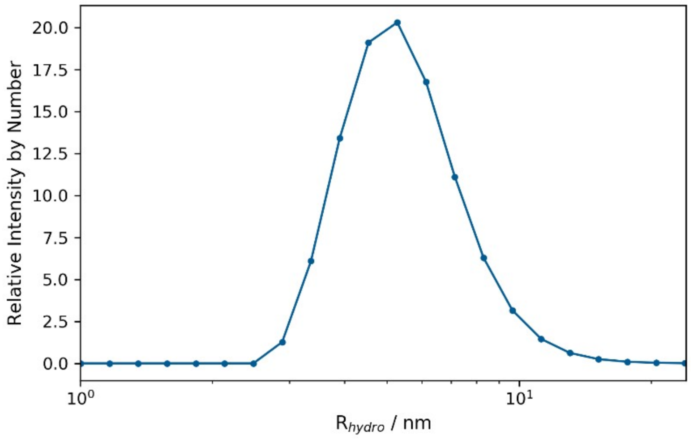

(**a**)

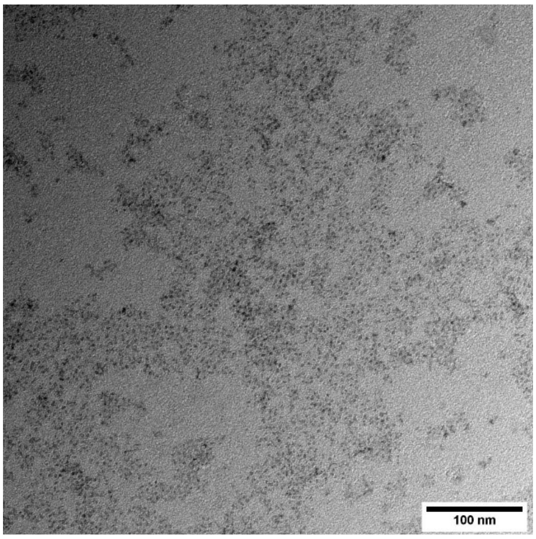

(**b**)

Figure S17. (**a**) Distribution of Stokes radii of *QDs* in toluene derived from light scattering experiments from five individual measurements. (**b**) TEM image of the corresponding *QDs* sample.
